# Artificial intelligence to enhance clinical value across the spectrum of cardiovascular healthcare

**DOI:** 10.1093/eurheartj/ehac758

**Published:** 2023-01-11

**Authors:** Simrat K Gill, Andreas Karwath, Hae-Won Uh, Victor Roth Cardoso, Zhujie Gu, Andrey Barsky, Luke Slater, Animesh Acharjee, Jinming Duan, Lorenzo Dall'Olio, Said el Bouhaddani, Saisakul Chernbumroong, Mary Stanbury, Sandra Haynes, Folkert W Asselbergs, Diederick E Grobbee, Marinus J C Eijkemans, Georgios V Gkoutos, Dipak Kotecha, BigData@Heart Consortium and the cardAIc group, Karina V Bunting, Otilia Tica, Alastair R Mobley, Xiaoxia Wang, Asgher Champsi, Nafeesah Ahmad Haider, Maximina Ventura, Alice Young, Paul McGreavy, Gastone Castellani, William Bradlow, Declan O'Regan, Julius Center

**Affiliations:** Institute of Cardiovascular Sciences, University of Birmingham, Vincent Drive, B15 2TT Birmingham, UK; Health Data Research UK Midlands, University Hospitals Birmingham NHS Foundation Trust, Birmingham, UK; Health Data Research UK Midlands, University Hospitals Birmingham NHS Foundation Trust, Birmingham, UK; Institute of Cancer and Genomic Sciences, University of Birmingham, Vincent Drive, B15 2TT Birmingham, UK; Julius Center for Health Sciences and Primary Care, University Medical Centre Utrecht, Utrecht, The Netherlands; Institute of Cardiovascular Sciences, University of Birmingham, Vincent Drive, B15 2TT Birmingham, UK; Health Data Research UK Midlands, University Hospitals Birmingham NHS Foundation Trust, Birmingham, UK; Institute of Cancer and Genomic Sciences, University of Birmingham, Vincent Drive, B15 2TT Birmingham, UK; Julius Center for Health Sciences and Primary Care, University Medical Centre Utrecht, Utrecht, The Netherlands; Health Data Research UK Midlands, University Hospitals Birmingham NHS Foundation Trust, Birmingham, UK; Institute of Cancer and Genomic Sciences, University of Birmingham, Vincent Drive, B15 2TT Birmingham, UK; Health Data Research UK Midlands, University Hospitals Birmingham NHS Foundation Trust, Birmingham, UK; Institute of Cancer and Genomic Sciences, University of Birmingham, Vincent Drive, B15 2TT Birmingham, UK; Health Data Research UK Midlands, University Hospitals Birmingham NHS Foundation Trust, Birmingham, UK; Institute of Cancer and Genomic Sciences, University of Birmingham, Vincent Drive, B15 2TT Birmingham, UK; School of Computer Science, University of Birmingham, Birmingham, UK; Alan Turing Institute, London, UK; Department of Physics and Astronomy, University of Bologna, Bologna, Italy; Julius Center for Health Sciences and Primary Care, University Medical Centre Utrecht, Utrecht, The Netherlands; Health Data Research UK Midlands, University Hospitals Birmingham NHS Foundation Trust, Birmingham, UK; Institute of Cancer and Genomic Sciences, University of Birmingham, Vincent Drive, B15 2TT Birmingham, UK; Patient and Public Involvement Team, Birmingham, UK; Patient and Public Involvement Team, Birmingham, UK; Amsterdam University Medical Center, Department of Cardiology, University of Amsterdam, Amsterdam, The Netherlands; Health Data Research UK and Institute of Health Informatics, University College London, London, UK; Julius Center for Health Sciences and Primary Care, University Medical Centre Utrecht, Utrecht, The Netherlands; Julius Center for Health Sciences and Primary Care, University Medical Centre Utrecht, Utrecht, The Netherlands; Health Data Research UK Midlands, University Hospitals Birmingham NHS Foundation Trust, Birmingham, UK; Institute of Cancer and Genomic Sciences, University of Birmingham, Vincent Drive, B15 2TT Birmingham, UK; Institute of Cardiovascular Sciences, University of Birmingham, Vincent Drive, B15 2TT Birmingham, UK; Health Data Research UK Midlands, University Hospitals Birmingham NHS Foundation Trust, Birmingham, UK; Department of Cardiology, Division Heart and Lungs, University Medical Center Utrecht, Utrecht University, Utrecht, The Netherlands

**Keywords:** Artificial intelligence, Healthcare, Management, Treatment

## Abstract

Artificial intelligence (AI) is increasingly being utilized in healthcare. This article provides clinicians and researchers with a step-wise foundation for high-value AI that can be applied to a variety of different data modalities. The aim is to improve the transparency and application of AI methods, with the potential to benefit patients in routine cardiovascular care. Following a clear research hypothesis, an AI-based workflow begins with data selection and pre-processing prior to analysis, with the type of data (structured, semi-structured, or unstructured) determining what type of pre-processing steps and machine-learning algorithms are required. Algorithmic and data validation should be performed to ensure the robustness of the chosen methodology, followed by an objective evaluation of performance. Seven case studies are provided to highlight the wide variety of data modalities and clinical questions that can benefit from modern AI techniques, with a focus on applying them to cardiovascular disease management.

Despite the growing use of AI, further education for healthcare workers, researchers, and the public are needed to aid understanding of how AI works and to close the existing gap in knowledge. In addition, issues regarding data access, sharing, and security must be addressed to ensure full engagement by patients and the public. The application of AI within healthcare provides an opportunity for clinicians to deliver a more personalized approach to medical care by accounting for confounders, interactions, and the rising prevalence of multi-morbidity.

## Graphical Abstract

**Graphical Abstract:**
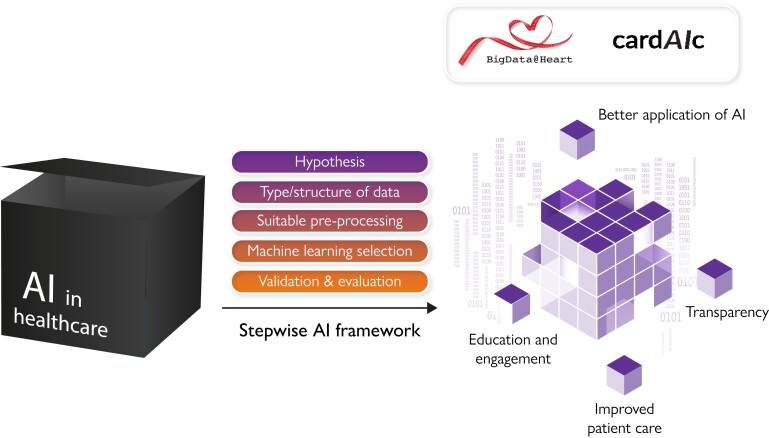
Step-wise workflow to improve the value of artificial intelligence in clinical research. AI, artificial intelligence.

## Introduction

Recent digital innovations provide an exciting prospect to improve the treatment, prevention, and prognostic evaluation of patients across the spectrum of healthcare. In cardiovascular disease (CVD), similar to other non-communicable diseases, the global number of deaths has increased by 31% from 1990 to 2010, despite a 21% reduction in age-standardized death rates in the same period.^1^ Complicating effective management, a quarter of CVD patients have five or more comorbidities, a four-fold increase from 2000 to 2014.^2^ New analytical and data-driven approaches could lead to a step-change in our understanding of multi-morbid patient groups, and open up the possibility of personalized therapeutic strategies.^3^

A wide range of data sources are now available for healthcare research, ranging from secondary/pooled analysis of clinical trials, to registries and electronic healthcare records (EHRs).^4,5^ Real-world data sets are often attractive due to their size, whereas data collected for research purposes are typically based on more selected populations, but with higher quality and less missing data.^6^ Artificial intelligence (AI) approaches have been developed in short order to deal with large or complex data sets,^3,7–10^ but a lack of transparency and understanding by health professionals has restricted their application and ability to impact patient management.

In this article, we summarize the *what* and *why* of applying AI techniques to health data, and then provide clear case examples of *how* such approaches can be performed on clinical data sets, leading to novel findings of relevance to routine practice. This framework for AI is designed to build a stronger foundation for collaboration between physicians and health data scientists, providing better understanding that can improve study design and clinical value. For further education and for readers with prior experience, we also provide the technical basis for the choice of machine-learning approach. The aim is to open up these new technologies and encourage widespread but appropriate use, whilst enhancing scrutiny and knowledge of their limitations in order to advance patient care.

## Workflow for the application of artificial intelligence techniques

The methods that underpin most AI applications in healthcare originate from the field of machine learning, which describes iterative learning by a computer algorithm that simulates (or betters) human intelligence. Healthcare applications have also benefited from progress made in other areas such as natural language processing and the integration of genomics and metabolomics (enriched data sets). A workflow for the application of AI techniques should start by questioning whether there are appropriate methodologies and data sets to address the research hypothesis. Conventional statistical analysis will often suffice; e.g. where mono-disease questions are raised and higher dimensional interactions are not anticipated.

**Figure 1:**
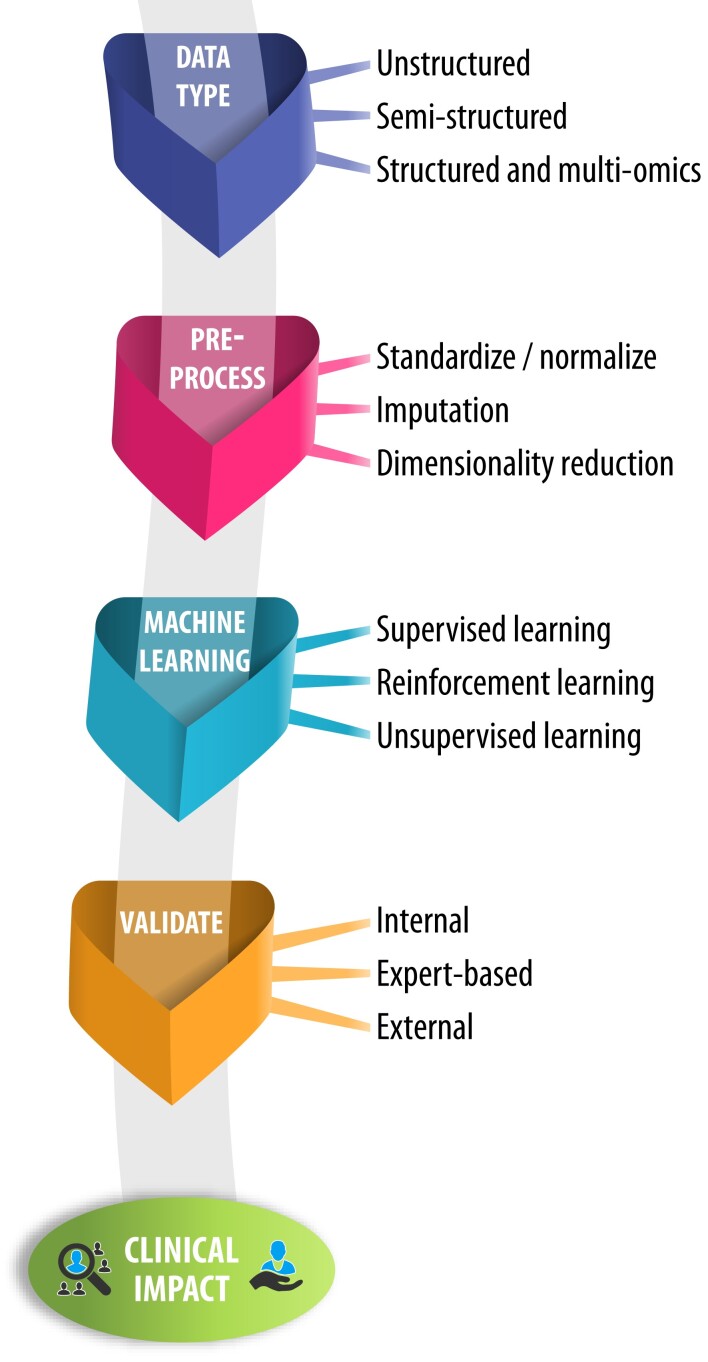
Artificial intelligence framework for clinical application. An overview of a framework to apply artificial intelligence, with standardized assessment and reporting of data acquisition, data pre-processing, and machine learning. These steps are interlinked with evaluation and validation to provide clinical value in real-world applications

**Figure 2:**
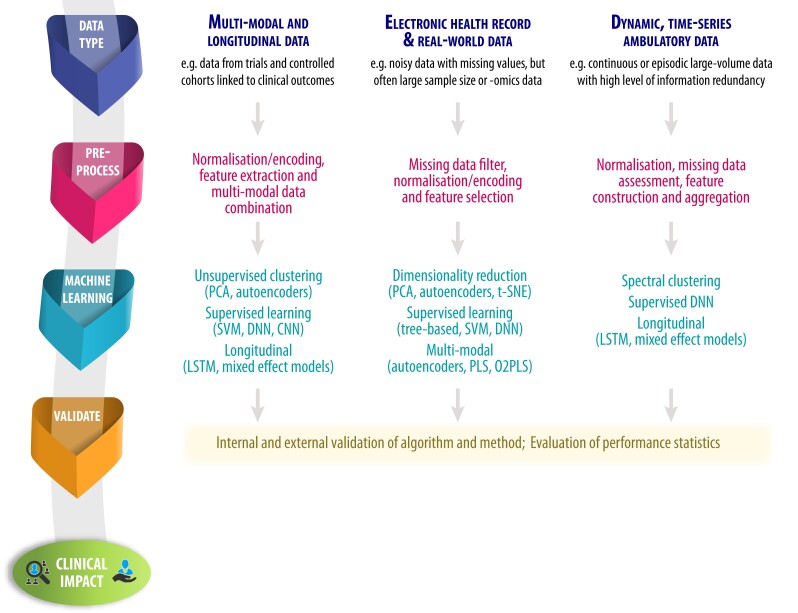
Examples of analytic steps based on data modality. Examples of pre-processing and machine-learning approaches based on the type of data available. CNN, convolutional neural network; DNN, deep neural network; LSTM, long short-term memory recurrent network; O2PLS, orthogonal two-way PLS; PCA, principal component analysis; PLS, partial least squares regression; SVM, support vector machine

An overall approach to the use of AI within healthcare is described in *Figure 1*. Following the development of a clear hypothesis, researchers (working with experienced data scientists) should first consider the type of data to be examined. This allows the research team to select computational algorithms based on the depth of data available (if granular and/or multi-modal), whether they are derived from noisy (but often large) data sets in the real-world, or if they are dynamic data such as wearable streams. To enhance research output, consider then in a step-wise fashion the aspects of data pre-processing, the desired approach to the algorithm employed for machine learning, and importantly, how findings will be evaluated and validated (see *Figure 2* for examples). For each step, the technical processes involved are detailed in the Supplementary material online, *Technical Appendix*.

### Step 1: type and collation of data

This first step will critically determine the design of the study, and facilitate appropriate analysis. No AI model is completely unbiased, and that begins at the point of data origin: *where and for what purpose the data were collected.* This question might already determine what underlying biases are present in the data and is not unique to an AI analysis; e.g. bias towards a certain ethnicity or age group. Furthermore, different classifications of data might be collected from different data modalities. This includes: (i) structured information such as demographics and blood biomarkers; (ii) semi-structured information, which includes text-based or continuously measured data such as time-series measurements from accelerometers (where raw data can be transformed into a sequence or graph structure^11^); and (iii) unstructured data, which include any type of imaging data. In the study design phase, the choice of data set is often determined by the research question; however, researchers should be aware that unstructured data may require more technical pre-processing. In terms of research output, the case studies highlight that structured data can be more readily interpreted to healthcare (e.g. Case 1), but unstructured data provide latent, hidden, or otherwise unknown relationships (e.g. Case 5). It can also be advantageous to use and link different types of data (as in Case 4). It is important to note that the linkage, i.e. the process of combining data for the same entities from different anonymized sources, is performed in a secure and privacy-preserving way. There are no boundaries for what is acceptable in terms of sample size, as this is dependent on the type of data (e.g. a wearables study may only have a dozen patients, but with millions of measurements for each patient over an extended period of time). Where there are only limited observations, AI techniques are likely to be of limited extra value. Another important consideration is the endpoints of the study, and whether these are well-defined, validated, or adjudicated.

### Step 2: pre-processing of data

Underlying biases in the data need to be explored, and then the data transformed into usable formats for machine-learning algorithms. The output of any AI model is only as good as its input; therefore, pre-processing is a critical step to plan a study and understand its findings. For numerical variables, this often means transforming values into a normal distribution (standardization) or bringing the values into a pre-defined range (normalization). Categorical data types are commonly encoded in a binary format. Missingness should be investigated, in particular if data are not missing at random, and then consideration of case deletion or imputation. Some data sets with a large number of variables may require a reduction in the dimensionality of the data (e.g. Case 2), either by linear approaches (such as principle component analysis) or by non-linear methods such as multi-dimensional scaling or neural network-based autoencoders. Other data types require different kinds of pre-processing: time-series data may be normalized by the capture period (e.g. Case 5); and images can be contrast-adjusted, scaled, or segmented (e.g. Case 7).

### Step 3: choosing the right machine-learning approach

This step assists with the choice of AI technique employed, determined by the clinical question or hypothesis, and the setting of how the machine will learn. In the supervised setting, the machine-learning approach uses labelled data to perform some form of prediction (e.g. length of stay, disease prognosis); in essence, it is using the human-derived output to train its prediction. In the unsupervised setting, the computational algorithms have no ground truth to compare with, so they are expected to infer relevant relationships between variables or with the stated outcome (e.g. define subphenotypes, patterns, or up/down-regulated gene expression).

A wide variety of machine-learning algorithms are now available. Decision Trees are constructed by splitting the training data iteratively until the data cannot be split further. Random Forests build models containing a number of Decision Trees, with each tree learned on a random subset of the data. Neural networks consist of multiple layers of computation, optimized to maximize predictive performance on a data set. Deep neural networks (DNN) use multiple additional layers to model complex non-linear relationships, with convolutional neural networks (CNN) being a specific class of DNN that provides feature selection during the learning process. Autoencoders learn a latent data representation of the original information via passage through a bottleneck layer. Additional machine-learning algorithms arise from the field of data integration, such as the two-way orthogonal partial least squares (O2PLS) approach, which decomposes two data sets into joint, orthogonal, and residual subspaces. The joint components capture the relationship between the data, while the orthogonal parts encode variation specific to each data set. Further advances, such as group sparse two-way orthogonal partial least squares (GO2PLS), can improve objectivity and interpretation.^12^ All of these AI approaches have been used in the healthcare setting, and with constant evolution, this list of resources will continue to expand.

### Step 4: validating and evaluating methods and results

Appropriate validation is needed in order to know how the study findings will apply to the real world. The importance of evaluating machine-learning output is no different from any other prediction task, and there remain challenges in generalizing from one data set to another, and then again to actual clinical practice. External validation should be the default approach for all AI studies, and usually involves estimating performance on completely unseen data. A gold-standard method is to apply a learned model to one or more data sets originating from a different cohort or study, preferably from a different site or time interval. Solely relying on internal validation methods is not advisable, although in some circumstances it may not be possible to share data or learn models. Options include *k*-fold cross-validation, in which the data are divided into distinct *k* subsets (folds), and each of the *k* subsets is used once for testing and the remaining (*k −* 1) subsets for training a model. The performance metrics on each fold can be aggregated to estimate the overall performance of the final model based on unseen data. To ensure an unbiased validation, each data point should be independent of any other data point, with data originating from the same patient in the same fold to avoid information leakage. An alternative to *k*-fold cross-validation is bootstrapping, which randomly splits the input data set into training and test sections many times with replacement in order to estimate model performance on unseen data and to construct confidence intervals. Overfitting of prediction models is a particular concern during the training stage of the machine-learning approach. For large data sets, the original data can be split into partitions (e.g. 70% for training and 30% to test), whereas for smaller data sets, full internal cross-validation is required. This enables researchers to select the best parameters internally without biasing further evaluation of the algorithm’s performance.

The evaluation of any machine-learning algorithm involves performance measures such as classification statistics, including true positive rate or sensitivity, true negative rate or specificity, and positive or negative predictive values. The F1 score (or Sørensen–Dice coefficient which is proportional to F1) is the harmonic mean of precision (positive predictive value in binary samples) and recall (sensitivity in binary samples) and is a measure of accuracy (correctly predicted cases compared with the complete number of samples in the data set). All these measures depend heavily on the underlying data set and the distribution of classes. A somewhat more objective measure is possible from receiver operating characteristic (ROC) analysis, in which the area under the curve expresses the performance of an approach regardless of the underlying class distribution. For time-to-event analyses, the same concordance probability measured by the ROC is calculated by the Harrel’s C-statistic. Both the F1 score and area under the ROC range from 0 to 1, with higher values indicating better performance.

The explainability of AI approaches is of substantial interest in healthcare. Commonly, AI approaches outperform human experts in specialized tasks, yet they do not give a reason for a particular prediction. Underlying biases in the data might lead to misclassification, and so caution must be used when interpreting AI approaches if they influence the care of patients.

## Case studies in the application of artificial intelligence techniques

The following case studies exemplify the potential clinical value of AI techniques. A summary of the case studies mapped to the AI workflow is presented in *Table 1*, providing worked examples across a variety of different data sources and data modalities that can aid interaction between clinical and data scientists. Patient and public involvement is critical to the development, community acceptance, and effective dissemination of research findings.^13^*Table 2* provides an insight into the use of AI in routine healthcare from a patient and public perspective.

**Table 1:** Overview of cases and mapping to artificial intelligence framework

Case	Data modality	Data origin	Learning setting and method	Number of participants	Validation	Key outcome from AI approach
1	Structured	Clinical trials	Unsupervised; autoencoders and clustering	*n* = 15 660 with HFrEF; 2837 with concomitant AF	Bootstrapping; leave-one-study-out cross-validation	Differentiation of patients based on interacting comorbidities and mortality benefit from beta-blocker therapy
2	Structured	Omics	Unsupervised; GO2PLS	Development cohort: *n* = 23; 13 with HCM. Validation cohort: *n* = 20; 10 with primary sclerosing cholangitis; 10 with ulcerative colitis	External validation	Integration of omic data sets to identify target genes and pathways for drug development and repurposing
3	Semi-structured	EHR (text)	Supervised; natural language processing	*n* = 3120; 1787 with HCM (17 199 letters)	Expert comparison; EHR coding comparison	Identification of patients that could benefit from therapies and specialist management
4	Unstructured	EHR (data)	Supervised; deep neural network	*n* = 65 565 patients with 137 018 electrocardiograms	Train-test split	Prediction of incident heart failure using routinely captured ECG data
5	Unstructured	Cohort within a clinical trial	Supervised; convolutional neural network	*n* = 41 with AF; wrist/smartphone devices with mean recording period 4.8 months (SD: 1.63)	Cross-validation	Approach to remotely monitor patients using wearable consumer devices
6	Unstructured	Observational cohort	Supervised; deep neural network	*n* = 4700; 1990s recordings of PPG	Train-test split; cross-validation; bootstrapping	Potential for effective screening of early vascular ageing using smartphones
7	Unstructured	Images	Supervised; convolutional neural network	*n* = 2480; 649 with PH; validation of segmentation: *n* = 831	Cross-validation; expert evaluation	Improved ability to determine patient prognosis based on cardiac imaging data

AI, artificial intelligence; EHR, electronic healthcare record; GO2PLS, group sparse orthogonal partial least squares; HCM, hypertrophic cardiomyopathy; PH, pulmonary hypertension; PPG, photoplethysmography.

**Table 2:** Patient and public perspectives on artificial intelligence

Thoughts about AI?	What are the challenges of implementing AI in healthcare?
• An exciting new approach that offers huge potential for better healthcare.	• Lack of understanding and training on AI among healthcare staff, and how it could support their clinical work.
• Could improve and speed up diagnosis, treatment, and referral processes.	• Cost of undertaking AI, with rapid technical advances resulting in equipment becoming obsolete quickly.
• Becoming commonplace in daily life but not as visible for healthcare.	• Large energy requirement for computer processing and lack of environmental awareness in line with developing public attitudes.
• Applying AI appropriately to health problems is a first important step, with the hope that useful AI can then be tested to improve the day-to-day care of patients.	• Need for transparent processes to ensure and demonstrate accuracy of data and reliability of algorithms.

Where are the opportunities for using AI in the healthcare setting?	What do patients and the public want and need before more widespread use of AI in healthcare?
• Better monitoring, awareness, and interest in healthcare issues, e.g. using consumer wearable devices.	• Better communication with the public is required to improve understanding of AI in healthcare, and ensure future engagement.
• Potential to reduce the need for patients to attend primary or secondary healthcare facilities.	• Reassurance regarding data security, in particular ownership, privacy, and potential sale of data to third parties and companies.
• Can leverage existing data sets without further direct patient involvement and ability to augment clinical decision-making.	• Technological developments must not accentuate existing health inequalities, but work to reduce them.
• Can help with unknowns by revealing information not immediately obvious to clinicians, which could improve treatments for patients.	• Clear explanation of how real-world use of AI can lead to benefits for patients and the healthcare system.
• To facilitate more efficient mass screening, reduce false positives and focus therapy on those at higher risk of poor outcomes.	

Written and compiled by the card*AI*c patient and public involvement and engagement team.

### Case study 1: treatment efficacy of beta-blockers using advanced clustering

#### Workflow

(i) Structured data from clinical trials; (ii) unsupervised clustering; (iii) internal and external validation by bootstrapping and leave-one-trial-out approach.

#### Rationale

Beta-blockers are highly effective in reducing mortality in patients with heart failure and reduced ejection fraction (HFrEF) in sinus rhythm, irrespective of patient age, or sex.^14^ However, the efficacy of beta-blockers was not demonstrated in patients with HFrEF and atrial fibrillation (AF), with a hazard ratio in this subgroup of 0.97 vs. placebo [95% confidence interval (CI): 0.83–1.14].^15^

#### Findings

**Figure 3:**
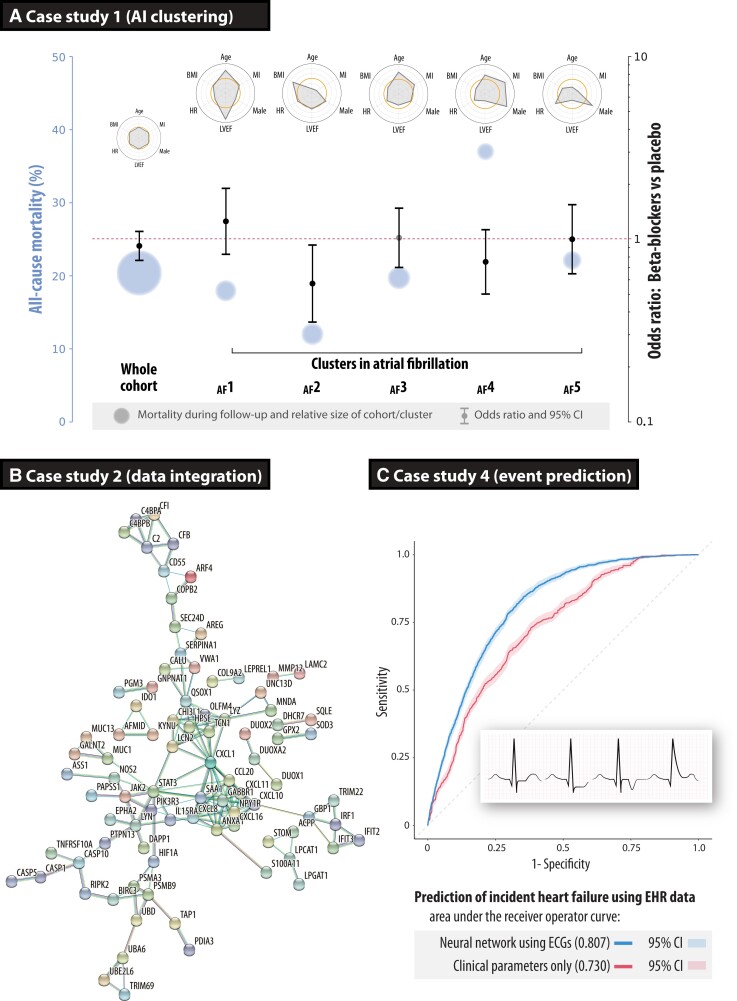
Case studies on how to improve phenotyping of patients. (*A*) Case study 1 using an artificial intelligence pipeline to cluster patients across nine clinical trials in patients with heart failure, reduced ejection fraction, and concomitant atrial fibrillation. Circles represent the average mortality risk (size relative to the number of patients in that cluster), with odds ratios for the efficacy of beta-blockers vs. placebo for all-cause mortality, and radar plots summarizing descriptors for each cluster compared with the cohort average (open orange circles at the centre of each radar plot). Reproduced and amended from.^3^ (*B*) Case study 2 showing the gene–gene interaction network of the top genes based on integrating transcriptomics and inflammation markers in patients with primary sclerosing cholangitis-inflammatory bowel disease and ulcerative colitis, externally validating the group sparse two-way orthogonal partial least squares approach.^12^ (*C*) Case study 4 depicting receiver operating characteristic curves for incident heart failure prediction in routine clinical practice, demonstrating superiority of the deep neural network model derived from digital electrocardiograms compared with clinical parameters (*P* < 0.00001). AF, atrial fibrillation; ECG, electrocardiogram; EHR, electronic healthcare records

Nine randomized trials provided individual patient data on 2837 HFrEF patients with an electrocardiogram (ECG) demonstrating AF at the time of randomization (see Supplementary material online, *Table S1* for additional methods). The median age was 65 years (IQR: 56–72), 24% women, and the baseline left ventricular ejection fraction was 27% (IQR: 21–33). All-cause mortality over a mean of 1.3 years of follow-up by intention to treat was not different between patients with AF randomized to beta-blockers or placebo (19.7% vs. 21.1%). Structured data underwent unsupervised AI clustering, with pre-processing using variational autoencoders as a dimensionality reduction algorithm. Four of five clusters showed a consistent lack of efficacy from beta-blockers. However, one cluster of younger AF patients did demonstrate a significant reduction in mortality with beta-blockers (odds ratio: 0.57, 95% CI: 0.35–0.93; *P* = 0.024; *Figure 3A*).^3^ Validation demonstrated good reliability and repeatability of results.

#### Critical interpretation

An unsupervised neural network-based approach was able to cluster patients with heart failure into different treatment groups based on their responses to beta-blocker therapy. Discovering novel patient subgroups may be valuable for future drug development and the design of clinical studies. As with any clinical trial, selection criteria can lead to differences with the real-world population, where additional patient heterogeneity could affect results.

### Case study 2: integration of high-dimensional omics data

#### Workflow

(i) Structured omics data; (ii) unsupervised—GO2PLS; (iii) external algorithm validation.

#### Rationale

The GO2PLS method was applied to integrate two heterogeneous omics data sets using simultaneous dimension reduction and feature selection. ChIP- and RNA-seq data from 13 patients with hypertrophic cardiomyopathy (HCM) and 10 controls were integrated (see Supplementary material online, *Table S2*), with better separation of cases and controls compared with conventional principal component analysis.^12^ GO2PLS was validated on a different data set with transcriptomics and inflammatory markers from mucosal samples taken from patients with primary sclerosing cholangitis-inflammatory bowel disease (PSC-IBD; *n* = 10) and ulcerative colitis (*n* = 10).^16^

#### Findings

The two sets of omics data were analysed; 1387 differentially expressed transcripts from RNA sequencing and seven inflammatory markers from flow cytometry. Using GO2PLS, the predicted sample scores showed a segregation of patients with PSC-IBD vs. ulcerative colitis. For the top selected genes, a functional association network was constructed using the STRING^17^ open-access database of known and predicted protein–protein interactions (*Figure 3B*). An enrichment analysis using the open-access DisGeNet^18^ gene-disease database was performed, and the genes selected by GO2PLS were significantly enriched for the IBD gene set (*P* < 0.0001, corrected for multiple testing). Additionally, hub genes responsible for the regulation of immune and inflammatory responses were found (chemokine ligand 1, CXCL1).^16^

#### Critical interpretation

Integration of different types of clinical data using machine learning, including genomics and proteomics, was an effective way of pinpointing target genes for new translational studies. Although based on small sample size and therefore limited in interpretation, the method used was validated by experiments on an external data set.

### Case study 3: application of text mining on healthcare data

#### Workflow

(i) Semi-structured data from EHR; (ii) supervised text mining; (iii) expert validation and EHR-coded data validation.

#### Rationale

Clinical notation is a rich source of information in the EHR, containing more detail on the health status of each patient than coded data alone. The challenge lies in appropriate extraction, validation, integration, and analysis of natural language.^19,20^ A supervised Komenti text-mining framework was used on clinical letters to identify patients with HCM not currently under specialist care, in addition to AF and heart failure status,^21^ and anticoagulant use.^22^ The approach was evaluated using a combination of expert manual validation and by comparing the derived cohort with coded EHR data (see Supplementary material online, *Table S3*).

#### Findings

The text-mining pipeline found 23 356 letters with keywords for HCM, of which 11 083 were relevant and described 3120 patients. The final classification determined 1753 real HCM cases, of which 333 patients had a positive family history, 357 had AF, and 205 had heart failure.^16^ Manual validation revealed an accuracy of 86.3% (95% CI: 82.3%–90.3%), with sensitivity against the structured EHR data of 86.5%. The 214 patients identified by the text-mining approach were not currently under specialist care. The accuracy of the anticoagulant prescription was 93.6% (95% CI: 88.6%–98.6%). When cross-checked against clinical records, 39 patients with AF were not anticoagulated and subsequently referred for treatment to prevent stroke and thromboembolism.^21^

#### Critical interpretation

Text-mining approaches can be used to identify patients with rare diseases, reducing the time, and effort required to discover patients, and enhancing their access to specialist management. This requires sufficient infrastructure and the ability to work securely on patient records, which in this case led to the optimization of patient treatment to improve clinical outcomes.

### Case study 4: predicting heart failure using electrocardiograms

#### Workflow

(i) Structured and unstructured data from EHR; (ii) supervised neural network; (iii) train-test split validation.

#### Rationale

Heart failure is a major public health concern, but there is limited scope to adequately identify those patients at risk who could benefit from preventative therapies. The 12-lead ECG encodes substantial information, of which only a small fraction is currently used in clinical practice. Within a large, secondary-care EHR, ECGs were linked to the incidence of heart failure determined by ICD-10 codes. A DNN model of digital ECG data was trained, optimized, and internally validated (see Supplementary material online, *Table S4*).

#### Findings

The analysis cohort consisted of 62 430 patients, with a median age of 60 years (IQR: 44–73), 48% women, and a median follow-up of 2.5 years (IQR: 0.7–4.5). The DNN model of 131 827 ECGs yielded an ROC area of 0.807 for incident heart failure (95% CI: 0.797–0.816; *Figure 3C*). This was superior (*P* < 0.0001) when compared with a model trained on clinical parameters alone (ROC: 0.730, 95% CI: 0.717–0.742). A combined ECG and clinical parameter DNN model yielded an ROC area of 0.826 for incident heart failure (95% CI: 0.816–0.836).

#### Critical interpretation

The application of neural network models to routine digital ECGs can identify patients at risk of developing heart failure. Although supported by internal validation, this approach requires external validation before implementation by clinicians to consider pre-emptive therapy to prevent heart failure and its substantial complications.

### Case study 5: integration of wearable data

#### Workflow

(i) Unstructured wearable sensor data; (ii) supervised neural network; (iii) cross-validation.

#### Rationale

**Figure 4:**
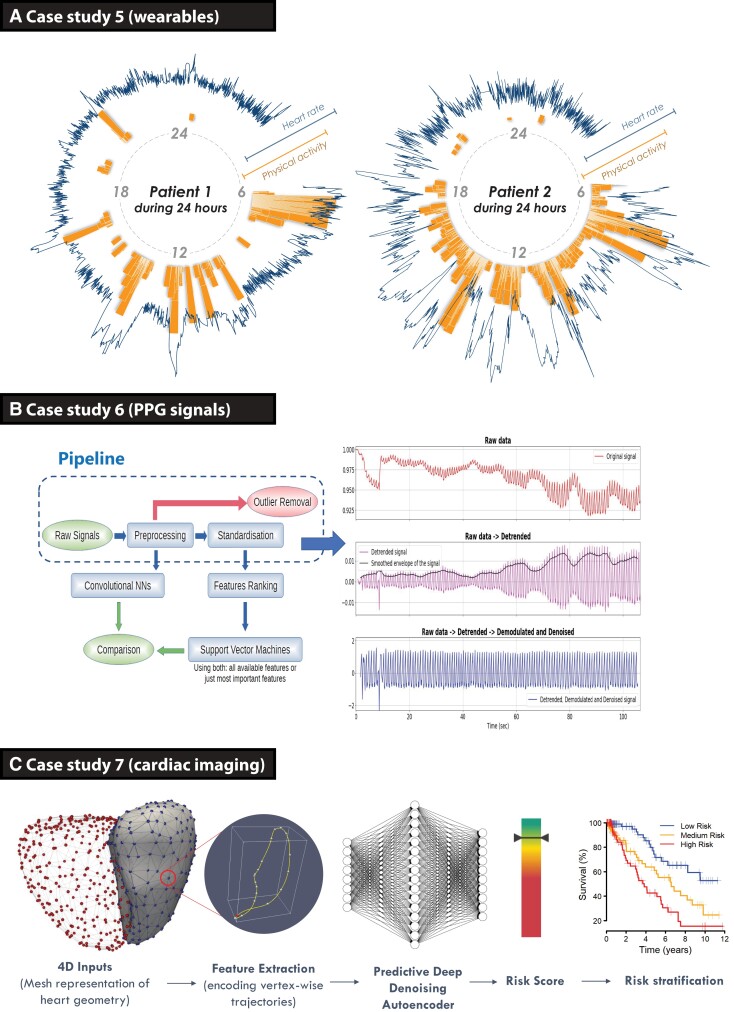
Case studies on how to incorporate novel investigations. (*A*) Case study 5 demonstrating ambulatory time-series data obtained from consumer wearable devices in two patients with atrial fibrillation. The blue line indicates minute-to-minute heart rate, in relation to the orange bars showing physical activity measured by step count over a 24 h period. Both patients had permanent atrial fibrillation and heart failure, the same level of symptoms (New York Heart Association Class III) and were treated with optimal medical therapy including heart rate control at the time of data capture (from the RATE-AF clinical trial).^23^ (*B*) Case study 6 showing the pipeline for signal processing of photoplethysmography signals using a smartphone camera, using deep learning (convolutional neural networks on pre-processed signals) and machine learning (features ranking and support vector machine on standardized features) to obtain clean signals for analysis and prediction of vascular ageing. (*C*) Case study 7 using dynamic neural network segmentation of volumetric cardiac magnetic resonance imaging to train a denoising autoencoder for stratification of survival in patients with pulmonary hypertension.^24^ AF, atrial fibrillation; CNN, convolutional neural network; HF, heart failure; PPG, photoplethysmography.

Monitoring of patients is typically limited to sporadic measurement during clinical care episodes, whereas wearable devices provide an opportunity for dynamic assessment in a range of environments. This study investigated whether a wrist-worn wearable device connected to a smartphone could provide equivalent information as traditional clinical trial assessments, tested within a randomized controlled trial of patients with AF and heart failure.^23^ Ambulatory heart rate and physical activity (step count) measurements were continuously collected from participants (*Figure 4A*), and compared with static measurement of heart rate on ECG and 6-min walk distance at trial visits to predict changes in New York Heart Association class. A CNN was developed using the wearable sensor data, consisting of a four-layer network with data augmentation and unsupervised pre-training to maximize generalizability (see Supplementary material online, *Table S5*). Performance was assessed by the F1 score and validated by leave-one-out cross-validation, with 95% CIs estimated by bootstrapping resampling.

#### Findings

Wearable data were collected from 41 patients randomized to either digoxin or beta-blockers, with 17 women (41%) and a mean age of 75.7 years [standard deviation (SD): 8.6]. The mean data collection period was 4.8 months (SD: 1.6). The CNN model using continuous wearable sensor data yielded an F1 score of 0.55 for prediction of New York Heart Association class at the end of the trial (95% CI: 0.40–0.70; with chance being 0.35). This was comparable with using a static measurement of 6-min walk distance and an ECG-derived heart rate at baseline, with an F1 score of 0.59 (95% CI: 0.44–0.74).

#### Critical interpretation

Scalable deep-learning frameworks have the capacity to remotely monitor patients with chronic conditions, after consideration of missing data and appropriate methods to deal with multi-channel time-series data. In this proof-of-concept study, continuously acquired wearable data provided equivalent information as conventional 6-min walk distance, showing the potential for consumer devices to replace inconvenient clinical tests.

### Case study 6: prediction of vascular ageing based on smartphone-acquired photoplethysmography

#### Workflow

(i) Unstructured photoplethysmography (PPG) sensor data; (ii) supervised neural network; (iii) train-test split, cross-validation, and bootstrapping.

#### Rationale

Smartphone-recorded PPG provides an opportunity for large-scale, non-invasive screening of cardiovascular conditions. Crowd-sourced PPG data from the ‘Heart for Heart’ initiative provided 90 s recordings from 4769 individuals. Simultaneous recordings of the red, green, and blue light spectrums obtained through PPG were pre-processed by de-trending, de-modulating, and denoising the raw signal. A peak detection algorithm was constructed, and low-quality signals were filtered. Machine-learning-based ridge penalized regression (RPR) was applied to extracted PPG features, and CNNs were applied to the whole PPG signal. Clinical features (sex, weight, height, and smoking status) with and without PPG data were compared in two groups according to their age, as a surrogate marker for vascular ageing (see Supplementary material online, *Table S6*).^25^

#### Findings

The 3612 participants had complete data, with a mean age of 49 years (SD: 15) and 1407 (39%) women. Raw PPG signals were successfully modulated (*Figure 4B*). The RPR approach selected two features (turning point ratio and the ‘a’ wave of its second derivative), whereas the best-performing CNN was a 12-layer ResNet. Comparable prediction performance was noted with both approaches. Using clinical features alone, the ROC area was 0.742 (95% CI: 0.635–0.841). ROC area increased to 0.947 with RPR by adding two PPG features in addition to clinical features (95% CI: 0.902–0.984). ROC area increased to 0.953 with the best CNN (95% CI: 0.916–0.989).

#### Critical interpretation

Computational approaches are able to isolate PPG signal features that are associated with age-related vascular changes. This case shows the potential for large-scale, person-led screening to identify individuals who could benefit from early therapy to prevent vascular dysfunction. Further work is required to evaluate beyond surrogate endpoints and test directly against clinical prognosis.

### Case study 7: using cardiac motion to predict survival in pulmonary hypertension

#### Workflow

(i) Unstructured geometrical data produced from cardiac magnetic resonance imaging; (ii) supervised neural network; (iii) expert, cross-validation, and bootstrapping.

#### Rationale

Pulmonary hypertension (PH) can lead to failure of right heart function and subsequent morbidity; however, the ability to accurately predict survival in PH is currently lacking. A deep CNN approach was developed to segment three-dimensional cardiac magnetic resonance imaging data into five regions (*Figure 4C*).^24,26^ The trained segmentation network was used to extract the right ventricular cavities acquired from 302 PH patients with known clinical outcomes (see Supplementary material online, *Table S7*). Segmentation accuracy was compared with semi-automated annotations and then quality-controlled by two experienced clinical experts.^27^ Survival prediction accuracy was tested using bootstrapped internal validation.^26^

#### Findings

The 649 patients with PH were included between 2004-2017, with a median follow-up of 371 days. The performance of the CNN-based segmentation model was evaluated on an independent set of 831 healthy subjects. Average Dice coefficients were excellent for each segment: 0.962 for the left ventricular cavity; 0.873 for the left ventricular wall; 0.923 for the right ventricular cavity; and 0.76 for the right ventricular wall. The average C-statistic for predicting survival outcomes using the CNN model was 0.75 (95% CI: 0.70–0.79), which was significantly higher (*P* < 0.005) when compared with a benchmark that uses conventional human-derived volumetric indices and clinical risk factors (C-statistic 0.59, 95% CI: 0.53–0.65). Segmentation results from 20 PH patients comparing automatic and manual measurements showed no significant difference between machine and human performance.

#### Critical interpretation

A supervised AI approach using CNN and autoencoders was able to accurately track heart motion on heart images, and predict survival in patients with PH. Similar to other studies where AI outperformed expert evaluation, the integration of such approaches into routine clinical practice is the next major challenge, plus critical elements of ongoing evaluation to ensure efficient clinical workflow and improved patient management.

## Discussion

This AI framework provides physician researchers with an understanding of how and when to apply AI algorithms to different types of healthcare data, and to encourage better collaboration with health data scientists. The step-wise approach starts from initial data selection and pre-processing, consideration of the right learning algorithm, and then evaluation and validation of results. To demonstrate potential impact, this framework was applied to a variety of data sources, ranging from structured data (clinical trials, omics, and patient records), semi-structured data (text mining), to unstructured data (time series, wearables, and imaging). Depending on the research question and data set, each data type required a different approach for pre-processing, learning methodology, and validation. The case studies highlight how the development in AI techniques, when applied correctly to clinical data sources, can achieve a step change in treatment selection, risk stratification, and patient well-being.

The application of AI in clinical medicine is an evolving technology with the potential to significantly expand knowledge and improve healthcare delivery. Multi-morbidity, and heterogeneous pathophysiology and clinical presentation that are commonplace in CVD limit traditional statistical methods, which struggle to include multi- and higher dimensional interactions between patient factors.^3,9,28,29^ Recent advances have also led to an increase in the potential application of AI-based methods, including large data volumes from EHR systems, registries, and trial collaborations,^6^ and complex data from wearable devices and body systems imaging.^9^ Using a data-driven approach, AI techniques can complement conventional statistical methods in a number of ways. For example, using neural networks to define novel phenotypes and treatment responses that can accelerate the drive towards personalized medicine.^3,30^ New text-mining approaches can be used to capture previously inaccessible information from clinical encounters, extracting the essence of the data into a machine-readable format for analysis while retaining depth and granularity.^7,22^ These natural language processing algorithms also present an exciting opportunity to validate outcomes from innovative EHR-embedded clinical trials.^31^ In the field of imaging, AI workflows can improve cost-efficiency by facilitating image acquisition and providing similar (or better) accuracy than human performance.^24,28,32^ Sensor data from consumer devices can be exploited by AI to digitize the process of remote data collection and disease monitoring, providing real-time data in the patient’s own environment,^33^ and contributing to disease prevention by effective screening.^25,34^ As with any emerging technique, the actual impact of AI is still being demonstrated, and this also applies to the case studies described. However, there is an encouraging example of AI deployment in the form of the first large, cluster-randomized trial of AI. Used on cardiac ECGs in 22 641 patients, the AI approach was able to improve the diagnosis of poor heart function.^35^ Although the absolute effect and impact were limited (diagnosis in 2.1% with AI vs. 1.6% for controls; *P* = 0.007), this trial demonstrates the applicability of AI techniques and also the rigour needed to test them. In the longer term, the evaluation of algorithms would benefit from routine benchmarking against current clinical practice, to understand the added value for clinical decision-making and healthcare workflow.

While recognizing the potential for AI-enhanced approaches, we should be mindful of underlying assumptions and limitations. Adequate pre-processing of data is vital so that any AI analysis has a robust basis for delivering new insight with predictions that make biological sense. Machine-learning algorithms, particularly in the field of deep learning, are often ‘black boxes’ *(Graphical Abstract)*where the action of computational processes is opaque.^36^ There is also an increasing gap in knowledge,^37^ with AI technology rapidly outpacing common knowledge among health researchers, clinicians, and the general public. This paper attempts to address some of these issues, but further structured education is needed across all stakeholders for a broader understanding about the use of AI algorithms in both clinical research and daily life. The lack of validation that is pervasive in the current literature is a major concern; in a systematic review of 82 studies using deep-learning algorithms, only 25 performed an external validation.^38^ This has led to new reporting guidelines for AI-based clinical trials (the CONSORT-AI extension),^39,40^ but gaps remain in the transparency of reporting. Researchers should be clear about their methods and be guided by the FAIR principles (Findability, Accessibility, Interoperability, and Reusability),^41^ so that other research groups can access data, provide independent validation, and ensure clinical value.

The social construct and licence for AI-based healthcare studies need specific consideration. In particular, researchers must carefully way up data privacy issues, consent (opt-in and opt-out approaches), the justification for data access, data sharing, and dissemination. These issues were recently highlighted by an international stakeholder group (CODE-EHR), providing a clear framework and checklist to encourage better use of structured healthcare data in clinical research.^42,43^ Strong patient and public engagement in the development and management of the research can be helpful to ensure these factors are embedded within the design of the study.^13^ Finally, while this article is designed to aid the application of novel AI techniques, the implementation of AI within routine healthcare remains in its infancy.^44^ Further work is needed (and ongoing) to ensure that challenges in implementation are addressed, including robust evaluation, governance, and acceptability to both care receivers and care givers.^45^

## Conclusion

A better and more focused application of AI has the potential to enhance stratification of patient risk and treatment response, aiding clinical decision-making and moving towards personalized therapeutic approaches. Appreciation is needed of the limitations of each data source and the different options for machine-learning algorithms, followed by detailed evaluation and validation. This approach is a foundation that can improve the precision and generalizability of AI to redefine complex disease phenotypes, discover new therapeutic targets, and enhance the delivery of healthcare for the public good.

### Key summary points

- AI approaches are widely used in healthcare, but their application varies and their methodology is often not transparent.
- A better use of AI for clinical research studies is limited by a lack of knowledge among all stakeholders, including clinicians, researchers, patients, and the public.
- A step-wise AI framework is defined to encourage improved application of these new technologies to a wider variety of clinical data.
- Case examples demonstrate how the integration of modern AI approaches can enhance the analysis of healthcare data, improve clinical decision-making, and subsequently lead to better outcomes for patients with CVD.

## Supplementary Material

ehac758_Supplementary_Data

## Data Availability

For information on the data and data availability underlying the case studies in this article, please see the references for each case study presented in Supplementary material online, *Tables S1–S7* in the appendix, with data shared on reasonable request to the corresponding author. The authors gratefully acknowledge incorporation of BEST trial research materials in case study 1, obtained through the National Heart Lung and Blood Institute (NHLBI) Biologic Specimen and Data Repository Information Coordinating Center (BioLINCC); the manuscript does not reflect the opinions or views of BEST or the NHLBI.
