# Supplementary material for: Artificial intelligence to enhance clinical value across the spectrum of cardiovascular healthcare
Source: Eur Heart J. 2023 Jan 11;44(9):713–25. doi: 10.1093/eurheartj/ehac758 (PMC9976986; doi:10.1093/eurheartj/ehac758)
Supplement: ehac758_Supplementary_Data [file ehac758_supplementary_data.docx]

**Title: Artificial intelligence to enhance clinical value across the spectrum of cardiovascular healthcare**

***Supplementary material***

[Technical appendix 2](#_Toc112331296)

[Step 1: Type and collation of data 2](#_Toc112331297)

[Step 2: Pre-processing of data 2](#_Toc112331298)

[Missingness 2](#_Toc112331299)

[Normalization and standardization 3](#_Toc112331300)

[Feature extraction and dimensionality reduction 3](#_Toc112331301)

[Anonymization 3](#_Toc112331302)

[Step 3: Choice of machine learning approaches 3](#_Toc112331303)

[Step 4: Validating and evaluating methods and results 4](#_Toc112331304)

[Resampling statistics (permutation test) and bootstrapping 4](#_Toc112331305)

[Biases and explainable artificial intelligence for health data science 5](#_Toc112331306)

[Example of an entire technical workflow 6](#_Toc112331307)

[Supplementary Table S1: Additional methods for case study 1 7](#_Toc112331308)

[Supplementary Table S2: Additional methods for case study 2 8](#_Toc112331309)

[Supplementary Table S3: Additional methods for case study 3 9](#_Toc112331310)

[Supplementary Table S4: Additional methods for case study 4 10](#_Toc112331311)

[Supplementary Table S5: Additional methods for case study 5 11](#_Toc112331312)

[Supplementary Table S6: Additional methods for case study 6 12](#_Toc112331313)

[Supplementary Table S7: Additional methods for case study 7 13](#_Toc112331314)

[References 14](#_Toc112331315)

# Technical appendix

## Step 1: Type and collation of data

When determining the design of an AI-based study, some basic technical issues should be considered. Here, we list several of the main issues and underlying considerations:

- Longitudinal or cross-sectional data
  - Is the collection type suited to answer the clinical question?
- Biases & dependencies
  - Are there underlying biases within the data or hidden dependencies between the collected data?
- Longitudinal data:
  - Is the longitudinal data selected in a time-based or event-based way?
- Real-world, clinical trials, or other:
  - Will the application of the model be outside of the collected data? For example, collected within clinical trials with an application in the real world.
- Multi-modalities:
  - For more than one data modality: are the different modalities collected in a consistent manner (devices, time, location, style of writing)?
- Linkage:
  - Is it possible to link from more than one data source in a secure and privacy-preserving way? (e.g., using Bloom filters or similar approaches^1^)
- Sample size:
  - The number of samples required for an analysis is dependent on the underlying question and features in the data. Furthermore, some algorithms require more samples by design.^2^

## Step 2: Pre-processing of data

To enable machine learning and AI approaches to analysis the collected data, it is essential to standardize and pre-process data. That includes, for example, removing inconsistencies and mapping to numerical data in case of categorical data, dealing with missing data, normalising numerical or image data, or extracting pre-defined features from the data and selecting subsets of features.

### Missingness

In clinical applications it is important to note that missingness of data can occur because of multiple reasons in the collection process. Generally, three types of missingness can be differentiated: data are missing completely at random (MCAR), missing at random (MAR), or missing not at random (MNAR). MCAR assumes that the missing data occurred from a completely random process, e.g. when an instrument lost connection to the network or it run out of batteries. MAR defines missingness within the other observed data, for example when a participant drops out of a clinical trial. In general, MNAR defines missingness which cannot be explained with the observed data, as the missing data might depend on the non-observed or collected data. Different approaches can be applied depending on the type of missingness, such as deletion (listwise or pairwise deletion) or imputation (simple substitution by average, or model-based imputation). Prominent examples of model-based imputation approaches are chained equations, support vector machines, autoencoders and generative adversarial networks.^3^

### Normalization and standardization

Commonly, data transformations are required to allow the AI or machine learning technique to avoid weighing some information more than others. This is particularly the case when the underlying algorithm is distance-based, as in the case of principal component analysis (PCA), k-nearest neighbours or neural networks. This requires transforming, or normalising, the original numerical feature data having the same numerical range. Prominent examples are standardization (or Z-score normalization) or the min-max normalization. Similarly, when using distance-based approaches, categorical data should be mapped into a numerical format. Often the so-called one-hot-encoding is employed, where each category is represented in a binary vector.^4^

### Feature extraction and dimensionality reduction

Other pre-processing approaches might only apply to continuous measured or image data, such as high or low pass filters, sliding window-based approaches, or frequency-based transformations like the Fast Fourier Transform (FFT). Similarly, high-dimensional data might require the reduction of the dimensions; this can be achieved by linear methods such as PCA, or non-linear approaches like kernel PCA, local linear embedding, autoencoders and others.^5^

### Anonymization

Another important aspect of pre-processing data lies in privacy-preserving anonymization. These range from lightweight pseudo-anonymization to more advanced approaches such as autoencoders, differential privacy and homomorphic encryption.^6^ Linking anonymized data in the multi-modal case can be challenging.

## Step 3: Choice of machine learning approaches

Depending on the type of data and mode, different kinds of machine learning approaches and methodologies might be employed. In general, if one or more outcome variables are known, supervised machine learning approaches are used. Supervised approaches are designed to build some form of model to predict a label. Labels can be manifold; they might consist of one or more binary or multi-class labels (in case of classification), or a numerical value or values (in case of regression). In contrast, unsupervised approaches are not provided with a label and are well suited to more explorative data analyses. They include approaches to find patterns in the data or to group similar examples into (potentially non-overlapping) clusters.^4^

Some of the most used supervised approaches are linear models, support vector machines, tree-based algorithms (including Random Forests and Boosted Decision Trees) and approaches employing artificial neural networks (convolutional neural nets [CNN] and deep learning). While all can be employed on well-structured data, neural network-based approaches have been favoured in recent years. There are, for example, CNN architectures for medical images, and recurrent neural networks (Long Short-Term Memory [LSTM]) models for multi-dimensional longitudinal data.^7^ Neural network-based approaches for text-based data have recently been overtaken in performance by attention-based architectures such as Bidirectional Encoder Representations from Transformers (BERT)^8^.

## Step 4: Validating and evaluating methods and results

When applying any AI or machine learning-based approach in healthcare, it is important to accurately evaluate its performance on future data. This might be its use within the same organisation or region, but also across different organisations, geographical areas or countries. Generally, validation approaches ensure the applicability of the produced models on previously unseen data. Validation approaches are commonly divided into internal and external validation. Internal validation is employed on subsets of the collected data, potentially even from the same study or organisation. Internal validation serves as a tool to verify that the model has been implemented correctly and does not suffer from overfitting. Common approaches for internal validation are simple train-test split, permutation-based approaches or randomised cross-validation. Within each of these validation approaches it is important to perform a secondary validation loop for (hyper)-parameter estimation.

External validation is employed when other datasets, trials or locations within the dataset exist to measure the applicability of the approach on data with different biases. For both validation types, the partition taken to estimate the performance of a model must be completely independent from any training data used in the model construction. This not only includes the actual model training, but also applies to the data pre-processing; otherwise so-called data leakage can occur. External validation can be made stronger if the data used for validation was separately collected (such as in the case of an additional follow-up study), or it originates from different studies after the building of the models (prospective and predictive validation).^9^

### Resampling statistics (permutation test) and bootstrapping

To test hypotheses and estimate confidence intervals, statistical methods are commonly employed when it can be assumed that the observed data originates from a well-known data distribution. However, in healthcare data this assumption is usually not fulfilled. Other statistical approaches based on randomization and resampling can be employed for cases where the distribution is unknown, or where a test for a known distribution is mathematically intractable.^10^ The two most common approaches are the permutation analysis and bootstrapping. In the permutation test, the null hypothesis assumes that the samples originate from the same distribution, i.e. that features and labels are independent and there is no, or only a small difference, in the performance of models trained on randomised or real label assignments. If this is not the case, the null hypothesis is rejected. More advanced approaches, such as applying independent randomization on the feature columns for each class exist.^11^ In general, permutation testing can be used to assess if an effect between the features and the labels/classes exists. Bootstrapping is well suited to estimate confidence intervals for the performance of an approach. Bootstrapping samples from the original data with replacement learns a model for each of the sampled datasets, and can be employed to estimate confidence intervals of the performance of the proposed approach. For both permutation testing and bootstrapping, the sampling is repeated a large number of times to produce statistically-significant results.

### Biases and explainable artificial intelligence for health data science

Employing AI and machine learning in healthcare poses a number of potential risks, such as privacy issues discussed previously. Another risk lies in the underlying bias and noise in the data collection in one environment, and the AI systems application in another. A prominent example can be seen in the application of the AI-based detection of diabetic retinopathy via an automated system, where a substantial number of assessments could not be performed due to differences in image quality.^12^ Furthermore, researchers and clinicians should be cautious about potential misclassification of the AI-based system. One potential approach to mitigate this risk is making these systems more transparent or interpretable.

The interpretability (or explainability) of approaches using artificial intelligence is of substantial interest in healthcare. Although AI approaches have been shown to outperform human experts in some specialized tasks, they commonly lack the reasoning for a particular prediction or assessment. From a technological perspective, several approaches exist to measure the importance of input features, regions within medical images or partitions in clinical letters, and are commonly derived indirectly. For tree-based approaches like Random Forests, the frequency of feature occurrences in the trees can be employed to create a feature importance ranking. Other model-specific approaches are available like layer-wise relevance propagation technique for Neural Networks or exBERT.^13^ In general, these approaches can be divided into model-level, i.e. considering the influence of features to the model, and prediction-level, i.e. considering the influence of features to a particular prediction instance. An example of a model-agnostic, prediction level strategy can be seen in the Shapley additive explanations (SHAP) approach^14^ that can be used to obtain the contributions of individual features for patient specific explanations. SHAP is based on so-called Shapley values inspired from cooperative game theory.^15^ The SHAP approach has been employed in AI approaches for the prediction of mortality of ICU patients^16^ and the importance of features for prediction of heart failure.^17^

## Example of an entire technical workflow


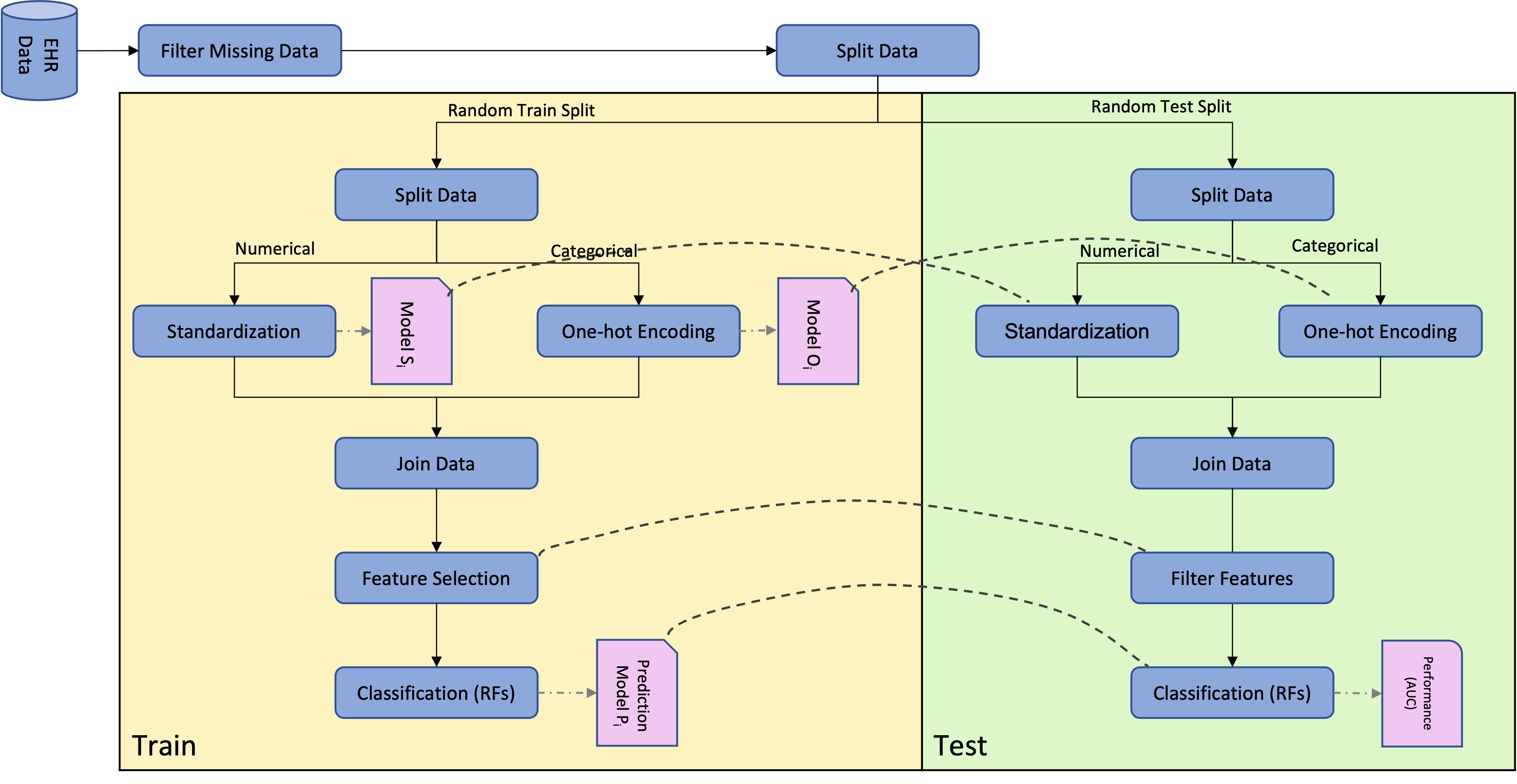


An example of a technical workflow. For simplicity, it only implements a train-test internal validation, not a cross validation. After data collection from an EHR system, samples with missing data is filtered out. The data is then split into a training and a test set. For a cross validation approach the test data would be one of the folds, while the training data would be the complete data without this fold. Using the training data only, the numerical data is standardized and the same standardization is applied to numerical data in the test data. Similarly, categorical data is encoded into binary vectors using one-hot-encoding. Both data types are then joined in the training. Potentially, features are selected and the selected information is employed to learn a classification model. This model is then applied to the normalised test data and its performance, e.g. the AUC, is recorded.

AUC = Area under the Receiver Operating Characteristics curve; EHR = Electronic healthcare record; RF = Random Forest (as an example of a machine learning algorithm).

## Supplementary Table S1: Additional methods for case study 1

| **Case** | Treatment efficacy of beta blockers using advanced clustering |
| --- | --- |
| **Data type** | Clinical trials |
| **Workflow** | (1) Structured data from clinical trials  (2) Unsupervised clustering  (3) Validation by evaluation of robustness and leave one out approach. |
| **Detailed methods** | Nine randomised controlled trials of beta-blockers versus placebo were included of patients with heart failure and reduced ejection fraction (HFrEF). Heart rhythm at baseline (sinus rhythm or atrial fibrillation [AF]) was determined using electrocardiography.  Variational autoencoders (VAEs) were used for dimensionality reduction and compared to conventional principal component analysis (PCA); the VAE approach performed significantly better in this dataset (average best gap statistic value 1.447 versus 0.812 for PCA; p=0.0001). The aim of the cluster analysis was to find similar subgroups within the cluster, yet dissimilar to other clusters. Hierarchical clustering and k-means++ were used as the underlying clustering techniques. The number of potential clusters were compared to dimensions and evaluated using three performance measures; the Silhouette Coefficient, the Variance Ratio Criterion and the Davies-Bouldin Criterion. Iterative repeated clustering was used to determine the number of clusters dimensions used and the model with the highest gap statistic value was selected.  Repeated clustering (*k*=100) of random subsets of the data (bootstrapping) was used to evaluate the robustness of the approach, and results were compared against random cluster assignments. The weighted Jaccard score was used to identify the overlap of clusters, and the Kolmogorov–Smirnov test for comparison to the random assignment. External validation was demonstrated using repeatedly generated clusters, with each iteration leaving one trial out. Using the generated cluster assignment as the target, random forest models were trained to predict cluster membership for each leave-one-trial-out dataset.^18^ |
| **Open access code** | (<https://pypi.org/project/hivae/>). |
| **Ethical approval** | All nine trials had appropriate ethical approval; this individual patient data meta-analysis used anonymised trial data under ethical approval 20122013-39 (University of East Anglia; 15 Jan 2013). |

## Supplementary Table S2: Additional methods for case study 2

| **Case** | Integration of high-dimensional omics data |
| --- | --- |
| **Data type** | Omics |
| **Workflow** | (1) Structured omics data  (2) Unsupervised – Group Sparse O2PLS  (3) Expert validation. |
| **Detailed methods** | We demonstrate GO2PLS for integration of transcriptomics and inflammatory markers from inflammatory bowel disease (IBD) samples: the mucosal samples of primary sclerosing cholangitis-inflammatory bowel disease (PSC-IBD, N=10) and from ulcerative colitis (UC, N=10). Two sets of omics data were analysed: 1387 differentially transcripts from RNA sequencing and 7 inflammatory markers from flow cytometry.  GO2PLS decomposes two omics datasets into joint, specific and residual components. The joint and specific components are linear combinations of the original variables. The joint loading values are estimated by maximizing the covariance of the projected omics data. The specific loadings are estimated by projections that maximize the variance of the residuals. A group penalty is imposed on the joint loadings to obtain sparse estimates that only involve a few variables. GO2PLS is implemented in the R package OmicsPLS on CRAN.  The predicted sample scores using GO2PLS showed segregation of the two groups (PSC-IBD vs. UC).^19, 20^ For the 200 top genes selected, we constructed the functional association networks using STRING database.^21^ We also conducted an enrichment analysis using DisGeNet - a gene-disease database based on data from text mining Medline abstracts using the BeFree System. Genes selected by GO2PLS were significantly enriched for the IBD gene-set (p<0.0001, corrected for multiple testing). Additionally, hub genes responsible for regulation of immune and inflammatory responses were found (chemokine ligand 1, CXCL1). |
| **Open access code** | <https://cran.r-project.org/package=OmicsPLS> |
| **Ethical approval** | University of Birmingham - Human Biomaterials Resource Centre [HTA Licence 12,358]; Mar 2012; ERN_13145 |

## Supplementary Table S3: Additional methods for case study 3

| **Case** | Application of text mining on healthcare data |
| --- | --- |
| **Data type** | Electronic health records (text) |
| **Workflow** | **(**1) Semi-structured data from electronic health records  (2) Supervised text-mining  (3) Expert validation and using electronic healthcare coding. |
| **Detailed methods** | The Komenti semantic text-mining framework was used to evaluate and extend the cohort of patients with hypertrophic cardiomyopathy (HCM) at University Hospitals Birmingham NHS Foundation Trust, which maintains a rare disease registry and specialist service for the disease.  The clinical text record was searched for mentions of HCM, using a vocabulary produced through a process of extraction from medical terminologies and on-site expert extension. Mentions were tokenised into sentences, then classified for negation, uncertainty, and family history status using a novel heuristic approach.  These sentence classifications were used to produce overall classifications for each patient described by the documents, to produce a cohort of HCM patients determined from clinical notes. We also classified patients for atrial fibrillation, heart failure, and HCM family history status.  Evaluation was performed with a combination of expert manual validation using a Komenti front-end interface, and by comparing the derived cohort with several structured resources at the hospital, including the rare disease registry and clinical coding system. In a separate text-mining orientated audit of anticoagulant treatments amongst patients already known to the registry, this information was extracted from clinical narratives using the same approach, and an expert manually evaluated the results using the Komenti tool.^22, 23^ |
| **Open access code** | <https://github.com/reality/Komenti>  <https://github.com/reality/komenti-validator> |
| **Ethical approval** | University of Birmingham - Ethical committee; 04 Mar 2020; ERN_20-0338. |

## Supplementary Table S4: Additional methods for case study 4

| **Case** | Predicting heart failure using electrocardiograms |
| --- | --- |
| **Data type** | Electronic health records (ECG data) |
| **Workflow** | (1) Structured and unstructured data from electronic healthcare records  (2) Supervised deep neural network (DNN)  (3) Train-test split validation |
| **Detailed methods** | All patients aged 18 and over with at least one digital 12-lead ECG sampled at 500 Hz recorded between December 2008 and March 2018 were included. Routine clinical data closest to the date of the ECG recording was collected from the electronic health record (EHR) system at University Hospitals Birmingham NHS Foundation Trust.  A DNN model based on the ECG leads (I,II and V1-6) was created using the Keras framework with Tensorflow (Google, Mountain View, USA). DNN models were developed using a high-performance server with 1 TB RAM and three P100 graphics cards (NVIDIA, Santa Clara, USA) with 16 GB of VRAM and parallel architecture training.  Data from the analysis cohort was split three ways; the training dataset contained 70% of the data, and the optimization and internal validation each contained 15%. The DNN ECG model was developed using the training dataset formed of residual blocks, with convolutions of different filter sizes on the temporal axis, followed by convolutions on the lead axis. During model training batches of randomly selected patients were included and positive cases oversampled.  To improve performance, different architectures were evaluated in the optimization set, and each was trained three times to ensure model robustness.  Three types of models were developed and compared: (1) a regression model utilising only clinical parameters from the EHR using logistic regression; (2) a DNN model constructed from the ECG recordings; and (3) the output from the DNN ECG model combined with clinical parameters in a logistic regression model. The clinical parameters were pre-specified as age, sex, body mass index (BMI) and EHR coding for hypertension, AF, coronary artery disease, chronic kidney disease, diabetes mellitus, and chronic obstructive airways disease. |
| **Open access code** | <https://github.com/gkoutos-group/postcode> |
| **Ethical approval** | London - Bromley Research Ethics Committee; 05 Nov 2020; ERN_ 20/PR/0659. |

## Supplementary Table S5: Additional methods for case study 5

| **Case** | Integration of wearable data |
| --- | --- |
| **Data type** | Observational cohort within a clinical trial |
| **Workflow** | (1) Unstructured wearable sensor data  (2) Unsupervised convolutional neural network (CNN)  (3) Cross validation. |
| **Detailed methods** | Sensor data was treated as a continuous two-channel time series and standardised. Heart rate was scaled to have zero mean and unit variance, whilst step count was normalised into the interval [0,1] to preserve the zero property of measurements. A third channel denoting data missingness was added to capture missing data dependencies without imputation. Patients had clinical and outcome data assessed at start and end visits in the trial, between which they wore their devices for continuous monitoring. Data from a one-week period at the end of the trial was held as a validation set. The remaining data was augmented and used to train an unsupervised CNN on a discrimination task. The augmentation created a secondary dataset where the heart-rate and step count channels were randomly permuted across patients.  The unsupervised CNN was trained to discriminate between real and permuted data, to learn the nonlinear relationship between sensor channels. This was a 1D CNN with 3 convolutional layers, with 8, 20 and 32 kernels respectively of size 21, and unit strides, followed by a global average pooling layer with dropout (0.5) prior to a fully-connected sigmoid classification head, trained on binary cross-entropy loss. Architectural hyperparameters were selected by grid search over validation accuracy on the unsupervised discrimination task. Training samples were 6-hour windows of sensor data, offset along 2-hour intervals for all patients, resulting in a total of 115,713 datapoints for the unsupervised task.  After training, the model was applied to the 6-hour window preceding clinical assessment of New York Heart Association (NYHA) heart failure functional class. The output of the global average pooling layer was used to compute 32 latent features for each patient, which comprised the predictor variables for the sensor-based regression model. The sensor model and the clinical assessment model were both L2-normalised logistic regression models predicting NYHA class at study end.^24^ The validation F1 score was calculated using leave-one-out cross validation, with 95% CI estimated by bootstrap resampling. |
| **Open access code** | <https://github.com/gkoutos-group/wearable_data_embedding> |
| **Ethical approval** | East Midlands - Derby Research Ethics Committee; 25 Nov 2019; ERN_16/EM/0178. |

## Supplementary Table S6: Additional methods for case study 6

| **Case** | Prediction of vascular ageing based on smartphone-acquired photoplethysmography (PPG) signals |
| --- | --- |
| **Data type** | Observational cohort |
| **Workflow** | (1) Semi-structured from electronic healthcare records  (2) Supervised convolutional neural networks (CNN)  (3) Train-test split external validation. |
| **Detailed methods** | Data pre-processing was performed in order to de-trend, de-modulate and de-noise the raw PPG signal. This was completed by subtracting a centred moving average from the raw PPG signal and dividing this by the envelope (the absolute value of the Hilbert transform of the signal). Subsequently a peak detection algorithm was constructed, and poor signals with a low-quality score representing incorrect demodulation and noise were filtered out. From the filtered data, 38 features were extracted and robustly standardised.  For the machine learning approach, ridge penalised regression, linear regression and logistic ridge regression were employed to select the most appropriate extracted PPG features (turning point ratio and the ‘a’ wave of its second derivative) for predicting healthy vascular ageing.^25^ Confidence intervals were generated using computed bootstrapping with a repetition of n=1000. A pseudo-outcome for vascular ageing was constructed by an extreme phenotype strategy, which covers both ends of the age range: subjects aged 18-38 years were allocated as healthy, those aged 60-70 years as unhealthy.  For the deep learning approach, several CNNs with whole signals and no extracted features were used as input. CNNs were trained, epoch by epoch, using two thirds of the training dataset. Their performance was validated using the remaining one third of the training set; for each hyperparameter, different values were tried and the best was determined. |
| **Open access code** | <https://github.com/Nico-Curti/cardio>  <https://github.com/LorenzoDallOlio/vascular-ageing> |
| **Ethical approval** | Anonymised crowd sourced dataset used |

## Supplementary Table S7: Additional methods for case study 7

| **Case** | Prediction of motion using cardiac magnetic imaging |
| --- | --- |
| **Data type** | Images |
| **Workflow** | (1) Unstructured geometrical data produced from cardiac magnetic resonance (CMR) images  (2) Supervised convolutional neural network (CNN)  (3) Expert, cross validation and bootstrapping. |
| **Detailed methods** | A CNN model was trained using 3D volumetric CMR images and the corresponding segmentation labels from end-diastolic (ED) and end-systolic frames. The heart was segmented into 5 regions: left ventricular cavity, right ventricular cavity (RVC), left ventricular wall, right ventricular wall and background. The trained CNN was used to extract RVC at ED, acquired from 302 patients with pulmonary hypertension (PH). For each patient, the RVC segmentation was co-registered to form a sequence as a dense motion model. These segmentations were used to perform a non-rigid registration using cardiac atlases built from >1,000 high-resolution CMR images using a 3D cine balanced steady-state free precession sequence. This approach produces accurate, high-resolution and anatomically smooth segmentations from input images with low resolution, thus preserving clinically important anatomical features.  Motion tracking was then performed for each subject using a B-spline free-form deformation image registration method, with a sparseness regularization term. The motion field estimate is represented by a displacement vector at each voxel and at each time frame. Temporal normalization was performed before motion estimation to ensure consistency across the cardiac cycle.^26^  A template surface mesh with 18,028 vertices built from these high-resolution images was warped onto the ED segmentation of each PH subject. With the motion fields estimated from each cine CMR image, the warped template mesh was then propagated across the whole cardiac cycle. These time-resolved 3D mesh models were then used as inputs to train a supervised denoising autoencoder (DAE), which is a full-connected network trained by a mean squared error reconstruction loss and Cox survival loss, with 6-fold cross-validation to tune the built-in hyperparameters.^27^ The DAE trained in this way effectively learns a task-specific latent code representation trained on observed outcome data, in this case yielding a representation optimised for survival prediction. |
| **Open access code** | <https://github.com/j-duan/4Dsegment>  <https://github.com/UK-Digital-Heart-Project/4Dsurvival> |
| **Ethical approval** | NRES Committee London South East; 10 Sep 2013; ERN_13/LO/0695. |

## REFERENCES

1. Schnell R, Bachteler T, Reiher J. Privacy-preserving record linkage using Bloom filters. *BMC Medical Informatics and Decision Making*. 2009;**9**:41

2. van der Ploeg T, Austin PC, Steyerberg EW. Modern modelling techniques are data hungry: a simulation study for predicting dichotomous endpoints. *BMC Medical Research Methodology*. 2014;**14**:137

3. Awan SE, Bennamoun M, Sohel F, Sanfilippo F, Dwivedi G. A reinforcement learning-based approach for imputing missing data. *Neural Computing and Applications*. 2022;**34**:9701-9716

4. Mitchell TM. *Machine learning, International Edition*. McGraw-Hill; 1997.

5. Jia W, Sun M, Lian J, Hou S. Feature dimensionality reduction: a review. *Complex & Intelligent Systems*. 2022;**8**:2663-2693

6. Boulemtafes A, Derhab A, Challal Y. A review of privacy-preserving techniques for deep learning. *Neurocomput.* 2020;**384**:21–45

7. Zhang L, Lu L, Wang X, Zhu RM, Bagheri M, Summers RM, et al. Spatio-Temporal Convolutional LSTMs for Tumor Growth Prediction by Learning 4D Longitudinal Patient Data. *IEEE Trans Med Imaging*. 2020;**39**:1114-1126

8. Devlin J, Chang M-W, Lee K, Toutanova K. BERT: Pre-training of Deep Bidirectional Transformers for Language Understanding. *CoRR*. 2018;**abs/1810.04805**

9. Witten IH, Frank E, Hall MA. *Data mining: practical machine learning tools and techniques, 3rd Edition*. Morgan Kaufmann, Elsevier; 2011.

10. Beran R. The Impact of the Bootstrap on Statistical Algorithms and Theory. *Statistical Science*. 2003;**18**:175-184, 10

11. Ojala M, Garriga GC. Permutation Tests for Studying Classifier Performance. *J. Mach. Learn. Res.* 2010;**11**:1833–1863

12. Heaven WD. Google’s medical AI was super accurate in a lab. Real life was a different story. [**https://www.technologyreview.com/2020/04/27/1000658/google-medical-ai-accurate-lab-real-life-clinic-covid-diabetes-retina-disease/**](https://www.technologyreview.com/2020/04/27/1000658/google-medical-ai-accurate-lab-real-life-clinic-covid-diabetes-retina-disease/) [18/08/2022]

13. Biecek P, Burzykowski T. *Explanatory Model Analysis*. Chapman and Hall/CRC; 2021.

14. Lundberg SM, Lee S-I. A Unified Approach to Interpreting Model Predictions. 2017:4765-4774

15. Shapley LS. A Value for n-Person Games. In: Harold William K, Albert William T, eds. *Contributions to the Theory of Games (AM-28), Volume II*. Princeton University Press; 1953:307-318.

16. Thorsen-Meyer HC, Nielsen AB, Nielsen AP, Kaas-Hansen BS, Toft P, Schierbeck J, et al. Dynamic and explainable machine learning prediction of mortality in patients in the intensive care unit: a retrospective study of high-frequency data in electronic patient records. *Lancet Digit Health*. 2020;**2**:e179-e191

17. Lu S, Chen R, Wei W, Belovsky M, Lu X. Understanding Heart Failure Patients EHR Clinical Features via SHAP Interpretation of Tree-Based Machine Learning Model Predictions. *AMIA Annu Symp Proc*. 2021;**2021**:813-822

18. Karwath A, Bunting KV, Gill SK, Tica O, Pendleton S, Aziz F, et al. Redefining beta-blocker response in heart failure patients with sinus rhythm and atrial fibrillation: a machine learning cluster analysis. *Lancet*. 2021;**398**:1427-1435

19. Gu Z, el Bouhaddani S, Pei J, Houwing-Duistermaat J, Uh H-W. Statistical integration of two omics datasets using GO2PLS. *BMC Bioinformatics*. 2021;**22**:131

20. Quraishi MN, Acharjee A, Beggs AD, Horniblow R, Tselepis C, Gkoutos G, et al. A Pilot Integrative Analysis of Colonic Gene Expression, Gut Microbiota, and Immune Infiltration in Primary Sclerosing Cholangitis-Inflammatory Bowel Disease: Association of Disease With Bile Acid Pathways. *Journal of Crohn's and Colitis*. 2020;**14**:935-947

21. Szklarczyk D, Gable AL, Nastou KC, Lyon D, Kirsch R, Pyysalo S, et al. The STRING database in 2021: customizable protein–protein networks, and functional characterization of user-uploaded gene/measurement sets. *Nucleic Acids Research*. 2020;**49**:D605-D612

22. Slater LT, Bradlow W, Hoehndorf R, Motti DF, Ball S, Gkoutos GV. Komenti: A semantic text mining framework. *bioRxiv*. 2020;10.1101/2020.08.04.233049:2020.08.04.233049

23. Slater LT, Bradlow W, Motti DF, Hoehndorf R, Ball S, Gkoutos GV. A fast, accurate, and generalisable heuristic-based negation detection algorithm for clinical text. *Comput Biol Med*. 2021;**130**:104216

24. Kotecha D, Bunting KV, Gill SK, Mehta S, Stanbury M, Jones JC, et al. Effect of Digoxin vs Bisoprolol for Heart Rate Control in Atrial Fibrillation on Patient-Reported Quality of Life: The RATE-AF Randomized Clinical Trial. *JAMA*. 2020;**324**:2497-2508

25. Dall’Olio L, Curti N, Remondini D, Safi Harb Y, Asselbergs FW, Castellani G, et al. Prediction of vascular aging based on smartphone acquired PPG signals. *Scientific Reports*. 2020;**10**:19756

26. Duan J, Bello G, Schlemper J, Bai W, Dawes TJW, Biffi C, et al. Automatic 3D Bi-Ventricular Segmentation of Cardiac Images by a Shape-Refined Multi- Task Deep Learning Approach. *IEEE Trans Med Imaging*. 2019;**38**:2151-2164

27. Bello GA, Dawes TJW, Duan J, Biffi C, de Marvao A, Howard L, et al. Deep learning cardiac motion analysis for human survival prediction. *Nat Mach Intell*. 2019;**1**:95-104
